# Increased Number and Distribution of Cerebral Microbleeds Is a Risk Factor for Cognitive Dysfunction in Hemodialysis Patients: A Longitudinal Study

**DOI:** 10.1097/MD.0000000000002974

**Published:** 2016-03-25

**Authors:** Chao Chai, Zhiye Wang, Linlin Fan, Mengjie Zhang, Zhiqiang Chu, Chao Zuo, Lei Liu, E. Mark Haacke, Wenmei Guo, Wen Shen, Shuang Xia

**Affiliations:** From the Department of Radiology, Tianjin First Central Hospital, Tianjin (CC, MZ, CZ, LL, WS, SX); Department of Radiology, Yuhuang Ding Hospital, Yantai, ShanDong (ZW); Department of Prophylactic Inoculation, Tianjin First Central Hospital, Tianjin (LF); Department of haemodialysis, Tianjin First Central Hospital, Tianjin (ZC); Department of Radiology, Wayne State University, Detroit, MI (EMH); and Department of Radiology, CNPC Central Hospital (WG), Langfang, China.

## Abstract

The aim of this study was to explore the risk factors associated with longitudinal changes in hemodialysis patients including the correlation between number and distribution of cerebral microbleeds (CMBs).

Sixty-one hemodialysis patients were enrolled in this prospective study. Twenty-eight patients had follow-up examinations with a mean interval of 24.79 ± 5.17 months. The number of CMBs was manually counted on susceptibility-weighted imaging. Subjects were divided into 2 groups with and without CMBs. In the CMB group, 8 of 33 patients did not have a mini-mental state examination (MMSE) because of blurred vision. Multiple logistic regression was used to investigate the risk factors for CMBs. Partial correlation was used to explore the correlation between the increased number of CMBs and the change of MMSE scores.

CMBs were seen in 33 (54%) hemodialysis patients. Both age and pre/postdialysis systolic blood pressure (SBP) positively correlated with CMBs. Serum iron (SI), and high-density lipoprotein cholesterol (HDL-c) negatively correlated with CMBs (all *P* < 0.05). Among 25 patients with CMBs and MMSE, 9 patients had scores <27, which was considered as subnormal and most CMBs in these patients were located in the brainstem and basal ganglia. Considering age and follow-up time as the co-confounding factors, the number of new CMBs over the 2 imaging time points negatively correlated with the change of MMSE scores (*r* = −0.673, *P* = 0.023).

The presence of new CMBs was a risk factor for cognitive dysfunction and the location of CMBs may be correlated with cognitive impairment. Both SI and HDL-c were protective factors for the CMBs. The risk factors for CMBs included age, pre- and postdialysis SBP.

## INTRODUCTION

Cerebral microbleeds (CMBs), are defined as small, round or oval homogeneous hypointense lesions in T_2_-weighted gradient-echo (GRE) MRI and susceptibility-weighted imaging (SWI).^1–3^ They are not commonly seen on conventional magnetic resonance imaging (cMRI) or computed tomography (CT) imaging. CMBs detected on SWI appear to correspond histologically to small focal collective areas of blood-breakdown products leaking from damaged fragile small vessels into adjacent brain tissues and are predictive of bleeding in some cerebrovascular diseases, such as cerebral amyloid angiopathy (CAA) and hypertensive vasculopathy.^4–6^ CMBs can be seen in the normal elderly population and the prevalence increases with age.^7,8^ It has also been reported that CMBs are related to ischemic and hemorrhagic stroke, intracerebral hemorrhage, Alzheimer disease (AD), head trauma etc.^9–12^ CMBs are more common in patients with recurrent stroke compared with those with first-ever stroke, suggesting a close relationship with progression of cerebrovascular disorders.^13^ CMBs can also result in cognitive impairment and brain atrophy, which severely affect the quality of life.^14,15^

Although haemodialysis (HD) is an effective way to clear the uremic toxins such as serum urea, serum creatinine for sustaining the life of patients with end-stage renal diseases (ESRD), it can also result in small cerebrovascular disorders, such as cerebral infarction, cerebral hemorrhage, and CMBs.^16–18^ It has been reported that cerebrovascular disease is one of the leading causes of death in patients with haemodialysis.^17^ In these cerebrovascular diseases, intracerebral hemorrhage, which can be predicted by CMBs, is more frequent and fatal.^19^ To predict the occurrence of intracerebral hemorrhage in patients with hemodialysis, it might be useful to identify the small cerebrovascular disorders, especially microbleeds. To date, there have been only 3 studies exploring the prevalence and clinical markers of CMBs in HD patients,^17,18,20^ and there has been only one longitudinal study about the effect of CMBs on developing stroke in HD patients.^21^ It has been reported that CMBs correlated with cognitive impairment in patients with transient ischemic attack (TIA) or ischemic stroke,^22^ whereas the effect of number of CMBs on cognitive impairment in HD patients has not been investigated by cross-sectional or longitudinal studies. In these 3 previous studies, there were some limitations: First, the CMBs were detected by T2^∗^weighted images at 0.5 T in 1 study and 1.5 T in the other 2 studies. T2^∗^ weighted image was shown to be less sensitive in detecting CMBs than SWI and led to an underestimation of prevalence and inaccurate clinical marker findings for CMBs.^23–25^ Goos et al^25^ found that SWI can identify nearly twice as many microbleeds as T2^∗^weighted image. Second, these 3 studies were cross-sectional design and longitude study was needed to perform to observe the temporal changes of CMBs and cognitive function. The purpose of the present study was: to explore the correlation between the newly increased CMBs and changes of MMSE scores in hemodialysis patients by a longitudinal study; to observe correlation between the distribution of CMBs and MMSE scores in a follow-up MRI; and to investigate the risk factors for the occurrence of CMBs in patients with hemodialysis.

## MATERIALS AND METHODS

### Subjects

This prospective study was approved by the medical ethics committee of our hospital (IRB approval number: 2016N0001LW). The MRI data from 64 right-handed patients with hemodialysis were acquired from November 2012 to September 2014. The informed consent was acquired from all subjects before their participation in the MRI examination. All the patients with hemodialysis were at chronic kidney disease (CKD) stage 5 (estimated glomerular filtration rate [eGFR] < 15 mL/min/1.73m^2^) according to the guidelines for patients with end-stage renal disease (ESRD).^26^ The inclusion criteria were: all the patients with hemodialysis were at CKD stage 5 (eGFR <15 mL/min/1.73m^2^); the age of patients was 18 years or above; all had hemodialysis 3 times weekly using the same device of Fresenius 4008S Haemodialysis System (Fresenius SE & Co. KGaA, Bad Homburg, Germany) in center for dialysis in Tianjin First Central Hospital; all had the same types of dialysis concentrates and membranes and dialysis dose for hemdialysis; all had the same anticoagulants and antithrombotic agent therapic regimens to reduce the risk of bleeding during the hemodialysis according to the guidelines for hemodialysis treatment^26,27^; each hemodialysis session took 4 hours to make sure there was adequate dialysis; and all the MRI data from HD patients were available including: T_1_WI, T_2_WI, and SWI. The exclusion criteria were: the patients had other associated disorders, such as traumatic brain injury, AD, previous obvious ischemic stroke and hemorrhagic stroke, infective disorders, genetic diseases, drug abuse, liver diseases, kidney transplantation, and neuropsychiatric diseases or epilepsy; the congenital structural abnormalities were seen in the routine MRI; and the quality of the images was not good enough to observe the brain abnormalities clearly. According to the inclusion and exclusion criteria, 3 patients with hemodialysis were excluded because of poor image quality. Finally, the remaining 61 HD patients (male 37 and female 24, age range 19–75 years, mean 47 ± 14 years) with average dialysis duration of 28 months (1–114 months) were enrolled. The etiology of patients with hemodialysis included: 21 patients with chronic glomerular nephritis, 11 with hypertension, 9 with nephropathy, 7 with polycystic kidney, 3 with glomerular amyloidosis, 2 with IgA nephropathy, 1 with urinary infection, 1 with drug impairment, 1 with systemic lupus erythenlatosus nephritis, 1 with acute glomerular nephritis, 1 with exposure to toxic substances, 2 with operation, and 1 with unknown etiology. The group with CMBs included 33 patients (male 22 and female 11, age range 21–75 years, mean 53 ± 13 years) with average dialysis duration of 30 months (1–75 months) and the group without CMBs included 28 patients (male 15 and female 13, age range 19–63 years, mean 40 ± 13 years) with average dialysis duration of 26 months (1.5–114 months).

To explore the longitudinal temporal changes of CMBs and MMSE scores, 61 patients were invited to do a follow-up examination. Out of 61 patients, 12 patients refused to undergo the second examination, 14 patients had renal transplantation, 1 patient died, and 6 patients went to other dialysis centers and were lost to follow-up. So 28 patients (male 18, female 10, mean age 51 ± 13 years, age range 23–66 years) underwent the follow-up brain MRI examinations with a mean interval of 25 ± 5 months (range 8–31 months) from the first MRI examination from September 2013 to September 2015.

### MRI Parameters

All MRI data were acquired using a 3.0T MRI Siemens Tim Trio system (Siemens Medical Systems, Erlangen, Germany) with an 8-channel head coil. The subjects were informed to keep their head motionless during the MRI examination. All axial sequences were positioned parallel to the anterior-posterior commissural (AC-PC) line as seen on the middle sagittal plane and the number of scanned slices covered the whole brain parenchyma. Conventional axial T_1_- and T_2_-weighted images were used to exclude other cerebral abnormalities. A 3D flow-compensated GRE sequence was applied to acquire SWI data. The SWI parameters were: TR/TE = 27/20 ms; 1 acquisition; field of view = 230 × 200 mm^2^; flip angle = 15 degree; receiver bandwidth = 120 Hz/pixel; voxel resolution = 0.5 × 0.5 × 2mm^3^; number of slices = 56; slice thickness = 2 mm, and acquisition time = 179 s.

The number of CMBs was manually counted by 2 experienced and trained neuroradiologists (CC with 5 years and ZMJ with 3 years of experience in neuroradiology, respectively), both of whom were blinded to the clinical information of patients. For the case with disagreement, they had a discussion to reach a consensus. The final number of CMBs was determined after the discussion. The identification of CMBs should meet all the following inclusion criteria: small round or ovoid (rather than linear) homogeneous hypointensity with a diameter of 2 to 10 mm^28^ on SWI, a clear paramagnetic dipole effect in the SWI filtered phase images and bright on susceptibility weighted imaging and mapping (SWIM); blooming effect on SWI; no hyperintensity on T_1_-weighted image or T_2_-weighted image to rule out cavernous hemangioma; at least half of the lesion was surrounded by brain parenchyma; the lesions should be differentiated from other microbleeds mimics, such as calcification, vascular flow voids, or iron deposition; and the patients had no history of traumatic diffuse axonal injury.

**FIGURE 1:**
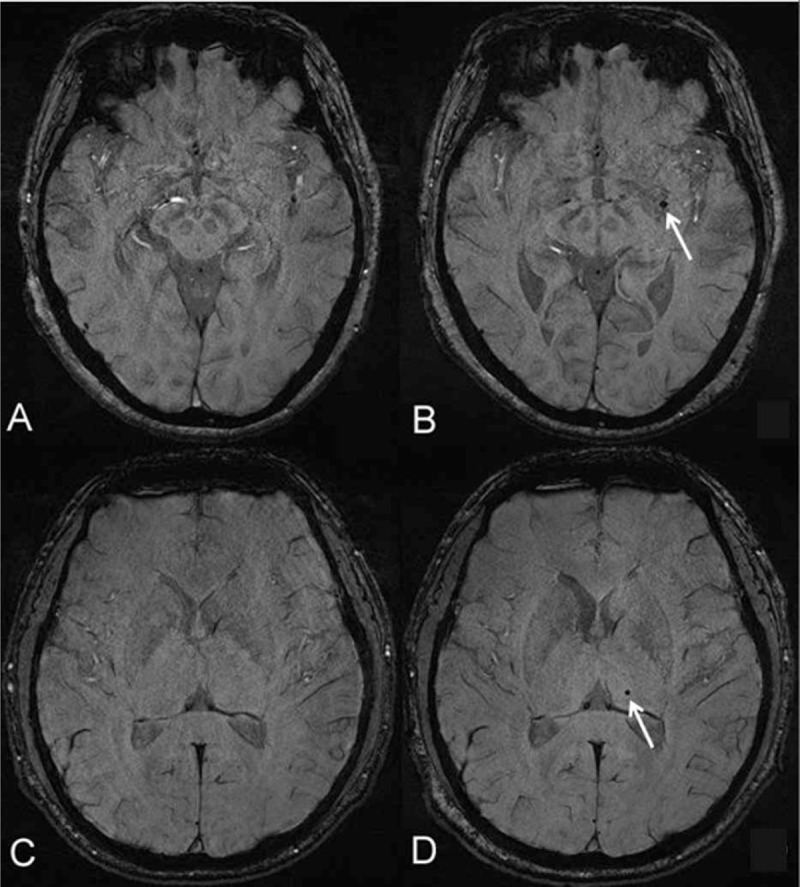
Temporal changes of cerebral microbleeds (CMBs) in patients with hemodialysis over a follow-up MRI. (A, B) A 64-year-old female patient with an interval 17 months of dialysis duration from the first MRI examination. There was a newly developed CMB (white arrow, B) in the left basal ganglia compared with the baseline examination at the same level (A). (C, D) A 30-year-old male patient with an interval 22 months of dialysis duration from the initial MRI examination. There was a newly developed CMB (white arrow, D) in the left dorsal thalamus compared with the baseline examination at the same level (C).

When 28 patients with hemodialysis finished their brain follow-up MRI examinations, the number of CMBs was counted and compared with the number at the initial examination by 1 experienced and trained neuroradiologist (CC with 5 years of experience in neuroradiology), who was blinded to the clinical data and the number of CMBs of patients at the initial examination (Figure 1).

### Laboratory Examination and Neuropsychological Evaluation

The age; dialysis duration; systolic blood pressure (SBP), diastolic blood pressure (DBP), and pulse pressure (PP) before and after hemodialysis; hemoglobin; serum urea; serum creatinine; serum albumin (ALB); white blood cell (WBC); alkaline phosphatase (ALP); triglyceride; total cholesterol (TC); high-density lipoprotein cholesterol (HDL-c); serum iron (SI); parathyroid hormone (PTH); and the presence or absence of diabetes mellitus (DM) and smoking were recorded. The above blood samples were acquired from the patients with hemodialysis within a week before MRI examination.

Ten patients with hemodialysis did not have the neuropsychiatric test because of blurred vision owing to the hemodialysis or primary renal disease at the initial examination. Finally, the remaining 51 HD patients with normal sight finished the Mini-Mental State Examination (MMSE) test on the same day before MRI examination in a quiet environment by the trained neuroradiologist (LL). To ensure reliable and valid results, the MMSE examination was performed according to the operating guidelines of MMSE.^29,30^ The MMSE is a neuropsychological test to evaluate global cognitive function and measures various domains of cognitive function including orientation to time and place; registration; concentration; short-term recall; naming familiar items; repeating a common expression; and the ability to read and follow written instructions, write a sentence, construct a diagram, and follow a 3-step verbal comman.^30^ The MMSE takes approximately 10 minutes to finish and the full potential score is 30 (see material, supplementary content, which demonstrates the **english version of MMSE scale**). Twenty-five of 51 HD patients had CMBs and 26 did not have CMBs. Twenty-seven patients with hemodialysis finished the MMSE test on the same day before the follow-up MRI examination in a quiet environment. One patient could not finish the MMSE test before both follow-up MRI and the first examination because of blurred vision.

### Statistical Analysis

The statistical analysis was performed using SPSS software package (version 16.0, SPSS Inc, Chicago, IL). Interobserver reliability between 2 trained neuroradiologists was evaluated using the intraclass correlation coefficient (ICC). The Kolmogorov-Smirnov test was used to explore the normality of the data. Because the ALP, neuropsychological score, and the number of CMBs of the patients with CMBs, the ALP, PTH, and dialysis duration of patients without CMBs were not normally distributed, the difference of the clinical factors (ALP, PTH, dialysis duration, and neuropsychological scores) between the 2 groups was explored using the Mann-Whitney test., the difference of the clinical factors (ALP, PTH, dialysis duration, and neuropsychological scores) between the 2 groups was explored using the Mann-Whitney test. The difference of other clinical factors including age; SBP, DBP, and PP before and after hemodialysis; hemoglobin; serum urea; serum creatinine; ALB, triglyceride; TC, HDL-c and SI between the 2 groups was explored using the 2 independent sample *t* test. The difference of categorical variables (sex, presence of smoking, and DM) between the 2 groups was explored using the *χ*^2^ test. Multiple logistic regression analysis was used to investigate the clinical factors for CMBs. According to the published studies, high ambulatory BP levels are important and possibly modifiable predictors for progression of CMBs.^31^ So 6 models including predialysis SBP, predialysis DBP, postdialysis SBP, postdialysis DBP, predialysis PP, post-dialysis PP were used, respectively, in the multiple logistic regression analysis to explore the effect of different blood pressures on the CMBs. Spearman rank correlation was used to explore the risk factors for the increased number of CMBs. Considering the age and follow-up time as the co-confounding factors, Partial correlation analysis was used to explore the correlation between the number of newly developed CMBs and the changes of MMSE scores. A *P* value <0.05 was considered significant.

## RESULTS

### Distribution Characteristics of CMBs and MMSE Scores

The number and distribution characteristics of CMBs in patients with hemodialysis are shown in Table 1. Out of 61 patients with hemodialysis, 33 patients had CMBs and the final total number of CMBs was 253 (range 1–110, mean 7.67 ± 18.96 per patient) manually counted by 2 experienced and trained neuroradiologists. The ICC between 2 trained neuroradiologists was 1.000 (95% confidence interval [CI] 0.999–1.000, *P* < 0.001). Twenty-five patients had MMSE tests in 33 hemodialysis patients with CMBs. Among 25 patients who had both CMBs and MMSE, 9 patients had scores <27, which was considered as subabnormal^32^ and most CMBs were located in the brain stem and basal ganglia (see table, supplementary Table 1, which demonstrates the distribution of CMBs and MMSE scores at the baseline examination).

**TABLE 1:**
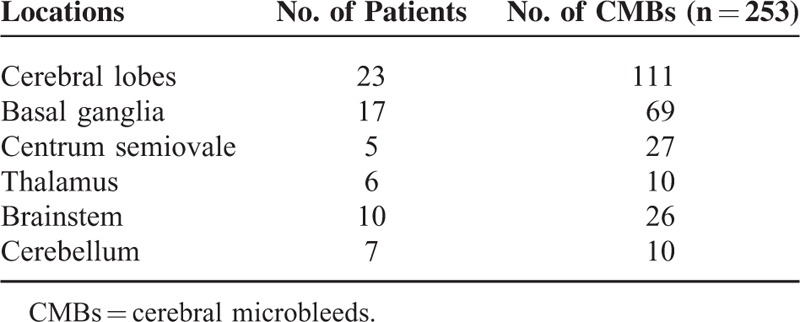
The Number and Distribution Characteristics of CMBs

### Comparison of Clinical Characteristics Between the 2 Groups

The comparison of clinical characteristics between with- and without-CMBs group is presented in Table 2. The age (*P* = 0.000), ALP (*P* = 0.018), pre- and post-dialysis SBP (*P* = 0.01, 0.04, respectively), and PP (*P* = 0.01, *P* = 0.009, respectively) of patients with CMBs was significantly higher than those of patients without CMBs. The SI (*P* = 0.03) and HDL-c (*P* = 0.02) of patients with CMBs were significantly lower than those of patients without CMBs. The MMSE scores of the patients with CMBs were significantly lower than those of the patients without CMBs (*P* = 0.02).

**TABLE 2:**
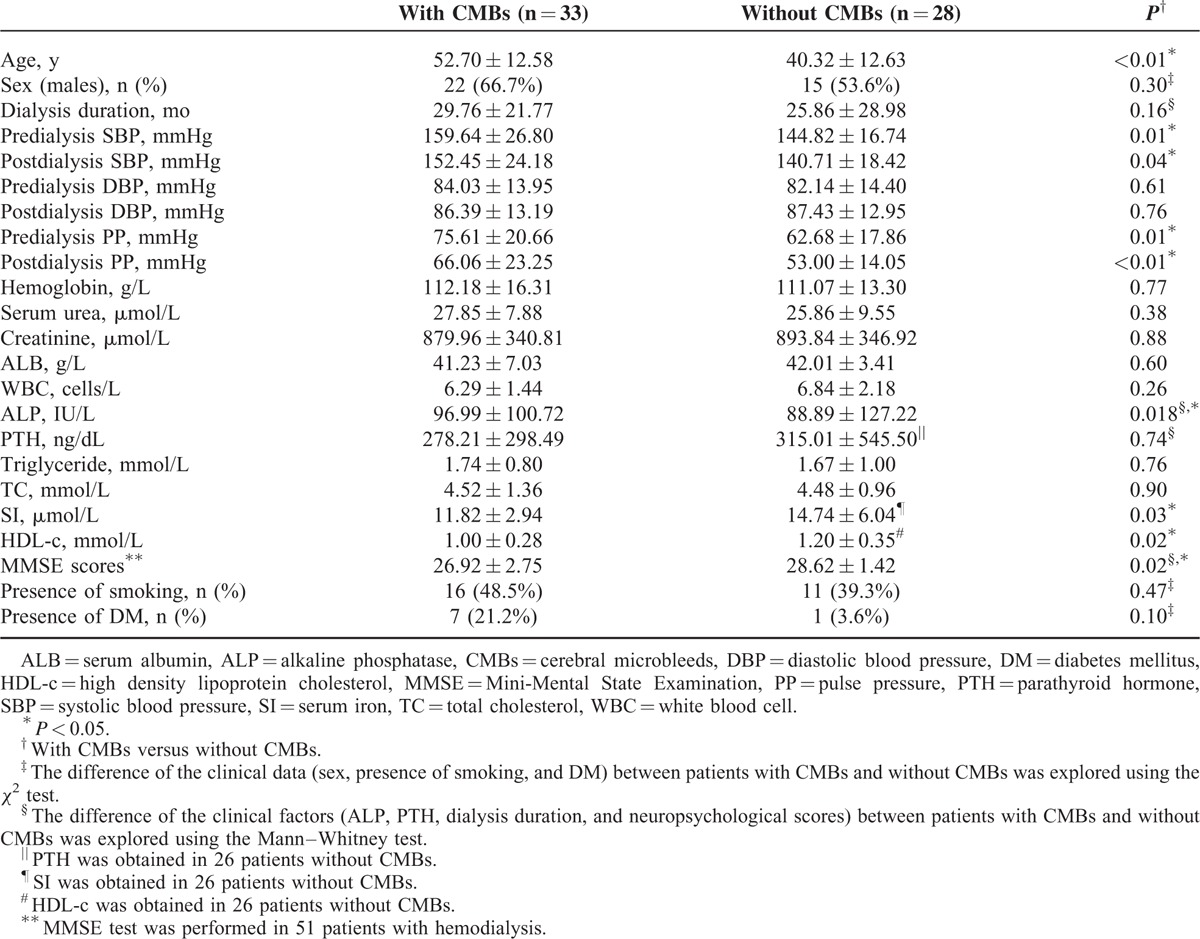
Comparison of Clinical Characteristics Between the Patients With and Without CMBs at Baseline

### Clinical Risk Factors for CMBs

According to a univariate logistic regression analysis, the clinical risk factors for CMBs included: age, HDL-c, SI, pre- and postdialysis SBP and PP (*P* = 0.001, 0.033, 0.032, 0.023, 0.048, 0.019, 0.020, respectively) (data not shown). In the multiple regression analysis, the clinical risk factors included age, pre- and postdialysis SBP, SI, and HDL-c (all *P* < 0.05) (Table 3). Age (model 1: *P* = 0.027, 95% CI: 1.011–1.198; model 3: *P* = 0.019, 95% CI: 1.018–1.225; model 4: *P* = 0.047, 95% CI:1.001–1.201; model 5: *P* = 0.040, 95% CI: 1.004–1.189, respectively), predialysis SBP (model 1: *P* = 0.041, 95% CI: 1.003–1.161) and postdialysis SBP (model 3: *P* = 0.027, 95% CI: 1.009–1.150) were positively correlated with the occurrence of CMBs and SI (model 3: *P* = 0.026, 95% CI: 0.516–0.959; model 4: *P* = 0.043, 95% CI: 0.567–0.991) and HDL-c (model 2: *P* = 0.044, 95% CI: 0.000–0.881; model 5: *P* = 0.047, 95% CI: 0.000–0.941; model 6: *P* = 0.038, 95% CI: 0.000–0.755) were negatively correlated with the occurrence of CMBs. Age was a risk factor for the increased number of CMBs among these above clinical factors in the cross-sectional study by Spearman rank correlation analysis (*P* = 0.013).

**TABLE 3:**
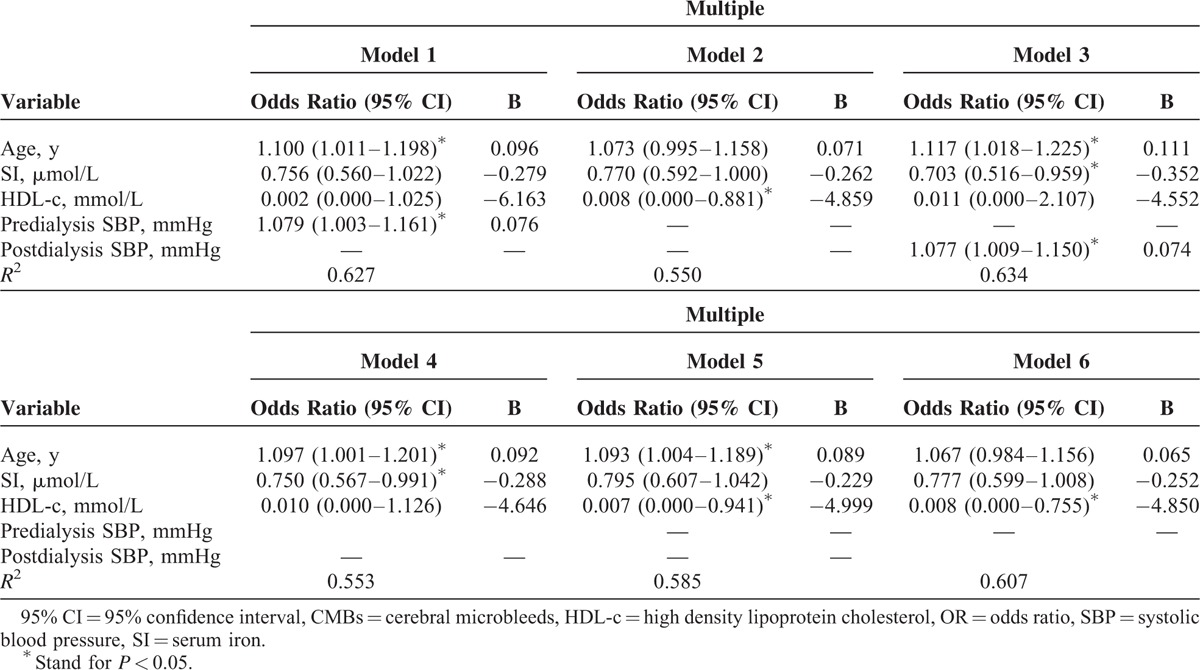
Risk Factors for CMBs Using Multiple Logistic Regression Analysis

### Temporal Changes of CMBs and MMSE Scores by Follow-up

The total number of CMBs of 28 patients with hemodialysis at follow-up was 162 (mean 5.8, range 0–25) and increased by 2.66-fold compared with the 61 CMBs (mean 2.2, range 0–11) at the initial examination. Out of 28 patients with follow-up, 14 (50%) patients had newly developed CMBs. The number of CMBs in the remaining 14 patients was not changed (**see table**, supplementary Table 2, which demonstrates the changes of CMBs number and MMSE scores between the baseline and follow-up). During the follow-up examination, MMSE scores of 16 patients decreased compared with those at the initial examination, MMSE scores of 7 patients were not changed and 4 patients had increased MMSE scores (see table, supplementary Table 2, which demonstrates the changes of CMBs number and MMSE scores between the baseline and follow-up).

### Correlation Between Increased Number of CMBs and Change of MMSE Scores

**FIGURE 2:**
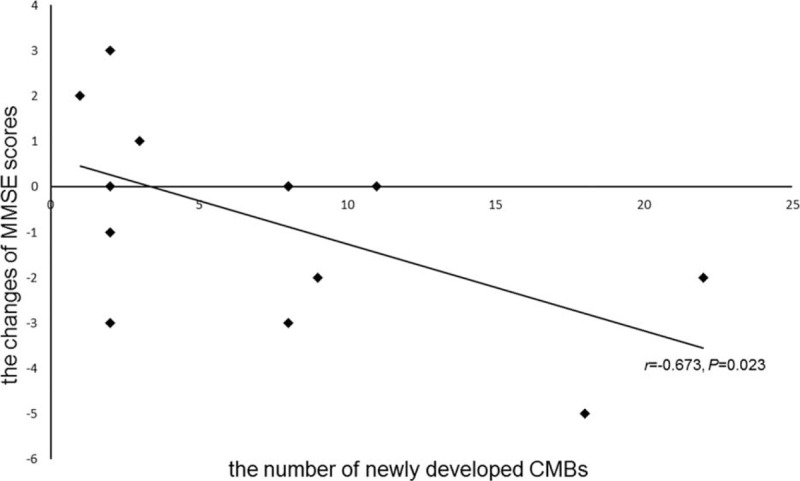
The correlation between the number of newly developed cerebral microbleeds (CMBs) and the change of MMSE scores. Considering age and follow-up time as the co-confounding factors, the number of newly developed CMBs showed a strongly negative correlation with the change of MMSE scores (*r* = −0.673, *P* = 0.023). Note: In our follow-up study, 14 patients had newly developed CMBs in the second examination. 13 of 14 patients had MMSE = Mini-Mental State Examination (MMSE) tests at the baseline and second examination and 1 patient did not have the 2 MMSE tests because of blurred vision. So we explored the correlation between the number of newly developed CMBs and the changes of MMSE scores in these 13 patients and the patient without MMSE tests at the baseline and follow-up was excluded. Two of 13 patients had the same increased number of CMBs (8) and the same changes of MMSE scores (0).

The correlation between the increased number of CMBs and the change of MMSE scores by a longitudinal study is demonstrated in Figure 2. Considering age and follow-up time as the co-confounding factors, the number of new CMBs was negatively correlated with the change of MMSE scores (*r* = −0.673, *P* = 0.023).

## DISCUSSION

Three important findings were found in our study. First, the presence of new CMBs was a risk factor for neurocognitive impairment in our longitudinal study, which was the first study to explore the effect of CMBs on cognitive function in hemodialysis patients by follow-up study. Second, the CMBs of patients with MMSE scores <27 mainly located in the brainstem and basal ganglia. These areas are considered critical brain structures for cognitive function. Third, the factors including age, pre- and postdialysis SBP were risk factors for CMBs. SI and HDL-c were both protective factors correlated with the occurrence of CMBs, which was not reported in previous studies.

In our follow-up study, we found the newly increased CMBs in 14 (50%) patients, which showed strongly negative correlated with the change of MMSE scores. In our study, the change of MMSE scores in hemodialysis patients with newly developed CMBs (mean follow-up time 23.23 months) was 0.77, which was higher than 0.2 reported in healthy people (mean follow-up time 24 months).^33^ The age of follow-up hemodialysis patients with newly developed CMBs in our study was 55.62 ± 10.71 years, which was less than 76.0 ± 5.1 years of healthy people,^33^ further suggesting that the newly developed CMBs was a risk factor for neurocognitive impairment in hemodialysis patients. During follow-up, the total number of CMBs was 162 and increased by 2.66-fold compared with the 61 CMBs in the same patients at the initial examination. The reason for the increased new CMBs may be that since haemodialysis cannot clear up all sizes and kinds of uremic toxins, the uncleared uremic toxins accumulated with dialysis duration and resulted in the more serious damage of small vessels and increased number of CMBs. It has been reported that the CMBs resulted in brain structural impairment because of inflammation and reactive oxygen species and the structural impairment finally causes the cognitive dysfunction.^34^ So the increased number of CMBs leads to more serious structural destruction and cognitive impairment, which were reflected by the decreased MMSE scores. In the follow-up patients, MMSE scores of 16 patients decreased at the second examination. Four patients with increased MMSE scores were found in the follow-up. The reason for this may be that the effect of hemodialysis on cognitive function is a dynamically fluctuating process and may result in cognitive impairment after a long dialysis duration.^35^ Although the hemodialysis may have dynamic effect on the cognitive function, which is a avoidless effect, Costa et al^35^ found that only a minority of patients exhibited significant individual cognitive fluctuations. In our study, the patients had hemodialysis 3 times weekly using the same device of Fresenius 4008S Haemodialysis System and each took 4 hours of haemodialysis session to make sure there was adequate dialysis according to the guidelines for hemodialysis, which reduced the harmful effect of haemodialysis on the cognitive function as far as possible.

Among the 25 patients with CMBs and MMSE at the first examination, 9 (36%) patients had scores <27, which was considered as subabnormal and most CMBs located in the brainstem and basal ganglia, indicating that the cognitive function may be affected by the distribution of CMBs. It has been reported that CMBs located in basal ganglia and frontal lobe have strong association with cognitive impairment.^32,36^ However, we did not further explore the effect of distribution of CMBs in the brain regions on the cognitive impairment because of the small sample size in each brain region. Future work should enlarge the sample size to further explore the correlation between the number of CMBs in the brain regions and the MMSE scores in a cross-sectional study and investigate in more detail the relationship between changes of cognitive function and the different locations of CMBs over a longer time using a longitudinal study.

In our study, the clinical factors including age, HDL-c, SI, pre- and postdialysis SBP, and PP were correlated with the occurrence of CMBs, which was basically consistent with the results of Naganuma et al's study. In the study of Naganuma et al's, they found that a high prevalence of CMBs occurred in patients with hemodialysis and the age and high blood pressure were strong factors associated with the occurrence of CMBs. In general, the occurrence of CMBs increases with aging, which is likely owing to advanced age-related small cerebrovascular diseases.^2^ We also found age was a risk factor for the increased number of CMBs. Hypertension has been reported to be the leading cause of microaneurysms and lipohyalinosis of small vessels, which can result in the rupture of small vessels and leakage of blood.^2^ The difference between our study and Naganuma et al's study was that we explored and found that HDL-c and SI were both protective factors correlated with the occurrence of CMBs in patients with hemodialysis, which were not included in previous studies.^17,18,20^ Kontush^37^reported that HDL-c had a wide range of atheroprotective functions, such as decreasing cellular death, diminishing vascular constriction, reducing inflammatory response, protecting from pathological oxidation, and combating bacterial infection et al. Gordon et al^38^ found that the elevated HDL-c was strongly correlated with decreased atherosclerotic events in large population-based studies. The protective function of SI for CMBs may be that: it has been documented that the patients with hemodialysis had iron-deficiency anemia, and iron-deficiency anemia can lead to cerebrovascular events.^39,40^ The supplement of serum iron can improve iron-deficiency anemia and protect the hemodialysis patients from cerebrovascular diseases. In the future, the protective mechanism of HDL-c and SI to prevent the occurrence of CMBs should be further investigated.

There were some limitations in our study. First, because the sample size of the longitudinal study is small, the correlation between newly developed CMBs and cognitive function needs further verification. Second, the number of CMBs was manually counted and may be a related bias for analysis. Third, all the patients in our study had same anticoagulants and antithrombotic agent therapic regimens according to the guidelines of hemodialysis, so we did not explore the effect of anticoagulant and antiplatelet therapy on the occurrence of CMBs.

In conclusion, our longitudinal study shows that the appearance of new CMBs was a risk factor for neurocognitive impairment. Furthermore, the location of CMBs may correlate with cognitive dysfunction. The HDL-c and SI were both protective factors correlated with the occurrence of CMBs in hemodialysis patients, which was also not found in the previous studies. Age and SBP were risk factors for the occurrence of CMBs.

## Supplementary Material

Supplemental Digital Content
