# Supplementary material for: Increased Number and Distribution of Cerebral Microbleeds Is a Risk Factor for Cognitive Dysfunction in Hemodialysis Patients: A Longitudinal Study
Source: Medicine (Baltimore). 2016 Mar 25;95(12):e2974. doi: 10.1097/MD.0000000000002974 (PMC4998366; doi:10.1097/MD.0000000000002974)
Supplement: Supplemental Digital Content [file medi-95-e2974-s001.doc]

**The title: Increased number and distribution of CMBs is a risk factor for cognitive dysfunction in haemodialysis patients: a longitudinal study**

**The first author: Chao Chai**

**Supplementary Table 1 Description of distribution of CMBs and MMSE scores at the baseline examination**

| Case | Age | MMSE | Brain stem | Basal ganglia | thalamus | Cerebral lobes | Centrum semiovale | Cerebellum |
| --- | --- | --- | --- | --- | --- | --- | --- | --- |
| Case 1 | 57 | 25* | 2 | 2 | - | - | - | - |
| Case 2 | 54 | 28 | - | 8 |  | 1 | 1 | 1 |
| Case 3 | 65 | 21* | 9 | 17 | - | 63 | 21 | - |
| Case 4 | 59 | 29 | - | - | - | - | - | 1 |
| Case 5 | 59 | 23* | - | 2 | - | 1 | - | - |
| Case 6 | 64 | 24* | - | 1 | - | 1 | - | - |
| Case 7 | 42 | 30 | - | 1 | 3 | 2 | - | - |
| Case 8 | 59 | 28 | - | - | - | 5 | - | - |
| Case 9 | 60 | 24* | - | - | - | 3 | - | 1 |
| Case 10 | 52 | 29 | - | - | - | 1 | - | - |
| Case 11 | 50 | 24* | 4 | - | - | - | - | - |
| Case 12 | 65 | 28 | 1 | 7 | - | 1 | - | - |
| Case 13 | 62 | 28 | - | - | 1 | - | - | - |
| Case 14 | 36 | 25* |  | 1 |  | 2 | - | - |
| Case 15 | 28 | 28 | - | - | - |  | - | 1 |
| Case 16 | 57 | 28 | 1 | - | - | 4 | - | - |
| Case 17 | 49 | 29 |  | - |  | - | - | 1 |
| Case 18 | 59 | 29 | - |  | 1 | 1 | - |  |
| Case 19 | 47 | 26* | - | 1 | - | - | - | 1 |
| Case 20 | 38 | 28 | - | 1 | - | - | - | - |
| Case 21 | 58 | 21* | 3 | 5 | - | 3 | - | - |
| Case 22 | 21 | 30 | - | - |  | 1 | - | - |
| Case 23 | 34 | 29 |  | - | - | 1 | - | - |
| Case 24 | 27 | 30 | - | - | - | 1 | - | - |
| Case 25 | 57 | 29 | 1 | 2 | - | 9 | 3 | 3 |

*MMSE scores less than 27.

**The title: Increased number and distribution of CMBs is a risk factor for cognitive dysfunction in haemodialysis patients: a longitudinal study**

**The first author: Chao Chai**

**Supplementary Table 2** Description of changes of CMBs number and MMSE scores between the baseline and follow-up

|  | Age (f) | Time  (m) | Number  (b) | MMSE  (b) | Number  (f) | MMSE  (f) | Number  (f - b) | MMSE  (f - b) |
| --- | --- | --- | --- | --- | --- | --- | --- | --- |
| Case 1 | 58 | 24 | 9 | 29 | 18 | 27 | 9 | -2 |
| Case 2 | 45 | 22 | 0 | 30 | 0 | 25 | 0 | -5 |
| Case 3 | 44 | 26 | 5 | 30 | 13 | 30 | 8 | 0 |
| Case 4 | 65 | 26 | 3 | 30 | 25 | 28 | 22 | -2 |
| Case 5 | 32 | 22 | 0 | 30 | 2 | 29 | 2 | -1 |
| Case 6 | 60 | 8 | 3 | 23 | 3 | 26 | 0 | 3 |
| Case 7 | 64 | 17 | 1 | 28 | 3 | 28 | 2 | 0 |
| Case 8 | 55 | 26 | 1 | 30 | 1 | 28 | 0 | -2 |
| Case 9 | 61 | 19 | 5 | 24 | 6 | 26 | 1 | 2 |
| Case 10 | 60 | 18 | 7 | 21 | 15 | 21 | 8 | 0 |
| Case 11 | 23 | 26 | 1 | 30 | 1 | 30 | 0 | 0 |
| Case 12 | 66 | 31 | 2 | 24 | 4 | 27 | 2 | 3 |
| Case 13 | 40 | 27 | 1 | 28 | 1 | 28 | 0 | 0 |
| Case 14 | 65 | 28 | 0 | 29 | 0 | 29 | 0 | 0 |
| Case 15 | 32 | 29 | 0 | 30 | 0 | 26 | 0 | -4 |
| Case 16 | 38 | 23 | 3 | 25 | 6 | 26 | 3 | 1 |
| Case 17 | 58 | 29 | 3 | 25 | 14 | 25 | 11 | 0 |
| Case 18 | 60 | 21 | 1 | 30 | 3 | 27 | 2 | -3 |
| Case 19 | 65 | 30 | 0 | 26 | 0 | 25 | 0 | -1 |
| Case 20 | 41 | 25 | 0 | 29 | 0 | 28 | 0 | -1 |
| Case 21 | 56 | 21 | 11 | 28 | 19 | 25 | 8 | -3 |
| Case 22 | 37 | 31 | 1 | 29 | 1 | 28 | 0 | -1 |
| Case 23 | 52 | 23 | 0 | 29 | 0 | 28 | 0 | -1 |
| Case 24 | 30 | 31 | 0 | 30 | 0 | 28 | 0 | -2 |
| Case 25 | 54 | 29 | 0 | 30 | 0 | 29 | 0 | -1 |
| Case 26 | 44 | 30 | 0 | 30 | 0 | 26 | 0 | -4 |
| Case 27 | 54 | 27 | 0 | - | 4 | - | 4 | - |
| Case 28 | 61 | 25 | 5 | 28 | 23 | 23 | 18 | -5 |
| Total |  |  | 61 | 755 | 162 | 726 | 101 | -29 |

f: follow-up; b: baseline; f-b: follow-up – baseline= follow-up minus baseline; MMSE= Mini-Mental State Examination.
